# Influence of Pentraxin 3 (PTX3) Genetic Variants on Myocardial Infarction Risk and PTX3 Plasma Levels

**DOI:** 10.1371/journal.pone.0053030

**Published:** 2012-12-28

**Authors:** Elisa Barbati, Claudia Specchia, Massimo Villella, Marco Luciano Rossi, Simona Barlera, Barbara Bottazzi, Luisa Crociati, Carmela d’Arienzo, Raffaele Fanelli, Cecilia Garlanda, Francesca Gori, Ruggiero Mango, Alberto Mantovani, Giuseppe Merla, Enrico B. Nicolis, Silvia Pietri, Patrizia Presbitero, Yukio Sudo, Alessandro Villella, Maria Grazia Franzosi

**Affiliations:** 1 Department of Inflammation and Immunology, Humanitas Clinical and Research Center, Rozzano, Milan, Italy; 2 Department of Translational Medicine, University of Milan, Milan, Italy; 3 Department of Cardiovascular Research, Istituto di Ricerche Farmacologiche “Mario Negri”, Milan, Italy; 4 Department of Biomedical Sciences and Biotechnologies, University of Brescia, Brescia, Italy; 5 Department of Cardiology, IRCCS Casa Sollievo della Sofferenza, San Giovanni Rotondo, Italy; 6 Department of Interventional Cardiology, Humanitas Clinical and Research Center, Rozzano, Milan, Italy; 7 Medical Genetics Unit, IRCCS Casa Sollievo della Sofferenza, San Giovanni Rotondo, Italy; 8 Perseus Proteomics Inc., Tokyo, Japan; 9 Department of Cardiology, F. Lastaria Hospital, Lucera, Italy; Leibniz-Institute for Arteriosclerosis Research at the University Muenster, Germany

## Abstract

PTX3 is a long pentraxin of the innate immune system produced by different cell types (mononuclear phagocytes, dendritic cells, fibroblasts and endothelial cells) at the inflammatory site. It appears to have a cardiovascular protective function by acting on the immune-inflammatory balance in the cardiovascular system. PTX3 plasma concentration is an independent predictor of mortality in patients with acute myocardial infarction (AMI) but the influence of *PTX3* genetic variants on PTX3 plasma concentration has been investigated very little and there is no information on the association between *PTX3* variations and AMI. Subjects of European origin (3245, 1751 AMI survivors and 1494 controls) were genotyped for three common *PTX3* polymorphisms (SNPs) (rs2305619, rs3816527, rs1840680). Genotype and allele frequencies of the three SNPs and the haplotype frequencies were compared for the two groups. None of the genotypes, alleles or haplotypes were significantly associated with the risk of AMI. However, analysis adjusted for age and sex indicated that the three *PTX3* SNPs and the corresponding haplotypes were significantly associated with different PTX3 plasma levels. There was also a significant association between PTX3 plasma concentrations and the risk of all-cause mortality at three years in AMI patients (OR 1.10, 95% CI: 1.01–1.20, p = 0.02). Our study showed that PTX3 plasma levels are influenced by three *PTX3* polymorphisms. Genetically determined high PTX3 levels do not influence the risk of AMI, suggesting that the PTX3 concentration itself is unlikely to be even a modest causal factor for AMI. Analysis also confirmed that PTX3 is a prognostic marker after AMI.

## Introduction

Coronary artery disease (CAD) is the most common clinical manifestation of cardiovascular pathology, the leading cause of mortality in the world [1], [2]. Its underlying process is atherosclerosis, a slowly progressive chronic disorder of large and medium-sized arteries [3]. Inflammation is considered a crucial component of atheroma development, progression and disruption [4].

Many of the (genes and) proteins implicated in CAD susceptibility, including pentraxins, are involved in innate and adaptive immunity [5], [6], [7]. On the basis of differences in the primary structure, pentraxins are divided into short pentraxins (C-reactive protein (CRP) and serum amyloid P (SAP) component), and long ones such as pentraxin-3 (PTX3) with a long N-terminal domain coupled to the C-terminal pentraxin domain [8]. PTX3 is produced and released by various cell types at the inflammatory site, including atherosclerotic lesions [9], [10], [11] and is involved in innate responses and regulation of inflammation [12], [13], [14].

The relationship with a marker of inflammation such as CRP prompted an examination of PTX3 plasma levels as a possible marker of human cardiovascular diseases, including atherosclerosis [9], angina pectoris [15], neointimal thickening after vascular injury [16] and heart failure [16], [17]. The general findings from these studies are the rapidity of its increase compared to CRP, the correlation with the severity of the disease and the lack of correlation between the levels of CRP and PTX3 [18], in line with the different regulation and cellular sources of the two molecules [8], [12]. In patients with acute myocardial infarction (AMI), PTX3 peaked in plasma sooner than CRP, 6–8 h from symptom onset [18] and, when measured with established markers including CRP, NT-proBNP and troponin-T, emerged as the only independent predictor of three-month mortality [19]. These and preclinical findings in mice [20], [21] suggest that PTX3 has a cardiovascular protective function by acting on the immune-inflammatory balance in the cardiovascular system.

Information on how *PTX3* genetic variants affect human diseases is restricted to infections [22], [23] and female fertility [24]. In the present study we explored the influence of *PTX3* genetic variants on the occurrence of AMI. In this multicentric genetic association study in a population of European origin we focused on the relations between three single nucleotide polymorphisms (SNPs) of the *PTX3* gene (rs2305619, rs3816527 and rs1840680), and plasma levels of the protein, the risk of AMI and its prognosis.

## Methods

### Ethics Statement

Written informed consent to participate in the study, including blood sampling, was obtained from each subject before entering the study. The investigation conforms to the principles outlined in the Declaration of Helsinki and was approved by the Institutional Review Boards of the participating hospitals (IRB Comitato Etico Scientifico IRCCS Casa Sollievo della Sofferenza, San Giovanni Rotondo and IRB Comitato Etico Humanitas Clinical and Research Center, IRCCS, Rozzano, Milan, Italy).

### Study Population

The study comprised 3245 unrelated subjects - 1751 AMI cases and 1494 controls - of European origin. Cases were patients with ST elevation AMI (1452 enrolled in the GISSI-Prevenzione (GISSI-P) trial [25] and 299 admitted to the Coronary Care Unit of the Humanitas Clinical and Research Center, in Milan, Italy). Controls were 1494 people without AMI, matched for age, selected from Italian biobanks in the participating Institutions (Mario Negri Institute, Casa Sollievo della Sofferenza Hospital, Humanitas Clinical and Research Center, and Lastaria Hospital). The study population can be considered representative of white Europeans in Italy, homogeneous in terms of genetic constitution.

### Genotyping

DNA was extracted from frozen EDTA-whole blood with a salting-out procedure [26]. The genotyping was carried out by the allelic discrimination assay using an ABI 7900 sequence detection system. Fluorescent data files for each plate were analyzed using the Sequence Detection System version 3.2 (Applied Biosystem). Real-time PCRs were done in 5 µl samples containing TaqMan Genotyping Master Mix, specific TaqMan® SNP genotyping assays purchased from Applied Biosystems (ABI, Foster, CA) and 10 ng of genomic DNA, according to the manufacturer’s instructions. To ensure the quality of automatic allele calling, all samples were analyzed in two replicates, and the concordance rate (between replicates) was 100%.

### PTX3 Assays

Plasma-EDTA PTX3 levels were measured by an enzyme–linked immunosorbent assay (ELISA) from Perseus Proteomics Inc. (Tokyo, Japan) [15] in a subset of 1455 subjects (708 AMI cases and 747 controls) who had whole-blood samples available. The lower limit of detection was 0.1 ng/mL; the inter- and intra-assay coefficients of variation were <4.3% and <4.1%, as described in the manufacturer’s instructions. In each plate, we measured PTX3 plasma levels of AMI cases and controls. In AMI patients, PTX3 plasma levels return to normal values within 125 hours (5 days) after the event [18]. Blood was therefore collected at the earliest 5 days after AMI event. Four AMI patients were excluded from the analysis since blood was sampled before that time. PTX3 levels from 76 controls (out of 362 with coronary angiography) were excluded from the analysis because blood was sampled too soon after angiography. PTX3 levels of seven controls were set to missing values because they were higher than the 99^th^ centile, 10 ng/mL in our sample. Therefore, 5% (87/1455) of PTX3 levels were excluded and the assay was done on 704 AMI cases and 664 controls.

### Statistical Analysis

Power analysis: the planned sample size of 1500 cases and 1500 controls enabled us to test for the association of three polymorphisms (overall alpha level 0.05) with the presence of the disease. Assuming that the prevalence of AMI in the Italian population (aged 35–75 years) is around 1% (www.cuore.iss.it/indicatori/prevalenza.asp), the frequency of the “at risk” allele is at least 35%, the genetic risk that we intended to detect, expressed in terms of odds ratio (OR), was at least 1.19. The calculated sample size guaranteed a power of 80% at a significance level of 0.017 for each SNP, analyzed with a 2-sided alternative hypothesis, assuming a log-additive model of inheritance. We used the software QUANTO 1.2 to calculate the sample size [27].

Continuous variables were compared among groups using Student’s T test, the Wilcoxon rank sum test or ANOVA, as appropriate. Differences in percentages were assessed by the Chi-square test. The pair-wise linkage disequilibrium (LD) among the three SNPs was assessed by the correlation coefficient R^2^. For each SNP, a Chi-square test was done to check whether the genotype frequencies were in Hardy-Weinberg equilibrium (HWE) among controls. HWE was tested using the genhw STATA package.

Frequency distributions by genotype and by allele were compared between AMI patients and controls. To quantify the effect of each SNP genotype on the risk of AMI and on PTX3 levels, we fitted a linear regression model, including age and sex as covariates. A codominant genetic model was tested. We examined the interaction of each SNP with the diagnosis of AMI on PTX3 levels by adding product terms to a multiple regression linear model, adjusted for age and sex.

Haplotype frequencies for rs2305619, rs3816527 and rs1840680 SNPs were calculated with the EM algorithm using UNPHASED 3.1 [28]. Rare haplotypes (frequency<0.05) were ignored. Haplotype frequency distribution was tested for the association with risk of AMI and with PTX3 levels.

We used a multivariable logistic regression model to determine, after adjustment for sex, age and other clinical covariates, the effect of PTX3 level on the risk of death, cardiovascular death and reinfarction among AMI patients. To ensure an overall type I error rate of 5%, a p-value<0.017 (i.e. 0.05/3) was considered significant for each of the three SNPs, according to the Bonferroni correction for multiple testing. Statistical analysis was done using STATA 9.0 (http://www.stata.com).

## Results

### Association between *PTX3* Genetic Variants and Risk of AMI

This study comprised 3245 subjects, 1751 AMI patients and 1494 controls (Table 1). Cases and controls were homogeneous in terms of age, but there were more males with AMI. As expected, controls had significantly higher HDL cholesterol and significantly lower triglycerides and LDL cholesterol. BMI did not differ in the two groups.

**Table 1:** Main clinical characteristics of AMI patients and controls.

		AMI (1751)	Controls (1494)	*p-value*
Sex	% male	81.8	71.8	*<0.001* **
**Age (years)**	Mean (SD)	58.1 (7.9)	58.3 (8.1)	*0.5* ***
**BMI (kg/m^2^)**	Median (IQR)	26.4 (24.3,29.0)	26.4 (24.2,28.9)	*0.4* *
**LDL Cholesterol (mg/dL)**	Median (IQR)	135 (113,157)	127 (105,150)	*0.0001* *
**HDL Cholesterol (mg/dL)**	Median (IQR)	40 (33,48)	54 (46,64)	*0.0001* *
**Triglycerides (mg/dL)**	Median (IQR)	145 (107,193)	107 (78,146)	*0.0001* *

* Wilcoxon rank sum test.

** Chi-square test.

*** T-test.

IQR, Inter quartile range.

**Figure 1:**
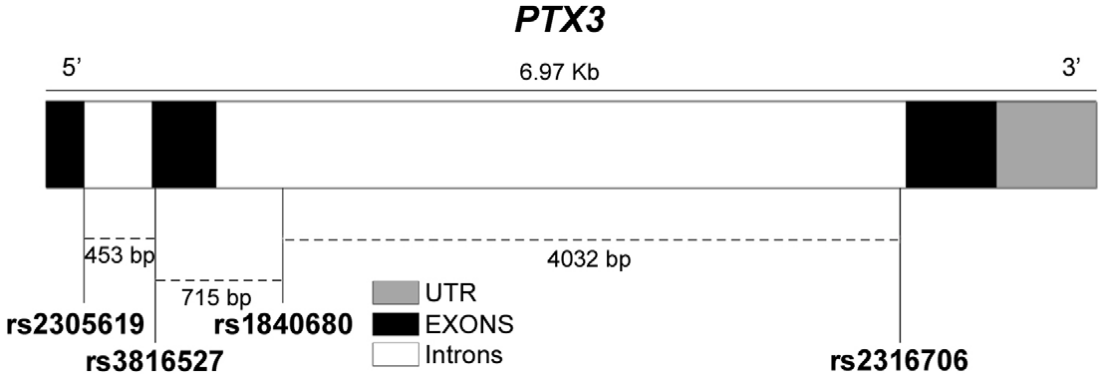
*PTX3* gene details. Gene map with position of SNPs typed and inter-marker distances.

We genotyped the study population for three *PTX3* SNPs, rs2305619, rs3816527 and rs1840680, that have been studied in association with susceptibility to *Mycobacterium tuberculosis* and *Pseudomonas aeruginosa* infections [22], [23], and fertility [24], but not with AMI. We also sequenced DNA samples from 50 cases and 50 controls to identify potential new *PTX3* variants. The sequencing of the *PTX3* coding region and intron/exon boundaries indicated only rs2305619, rs3816527 and rs2316706. The intronic rs2305619 and rs2316706 were in complete LD (R^2^ = 1). rs3816527 lies in exon 2 and is responsible for an aminoacid substitution in position 48 of the PTX3 primary structure (Asp48Ala). We included rs1840680 in line with previous association studies on *PTX3* genetic variants, but it was too far from the intron/exon boundaries to be revealed by sequencing. Hence, the large-scale genotyping was conducted on rs2305619, rs3816527 and rs1840680 polymorphisms. Figure 1 shows the *PTX3* gene organization and the position of the polymorphisms and Table 2 reports information about the three SNPs. Genotype frequencies were in HWE among controls (p = 0.97, p = 0.32, p = 0.98 for rs2305619, rs3816527 and rs1840680, respectively). The minor allele frequencies were similar to the frequencies reported in HapMap. The rs3816527 and rs1840680 SNPs were closely correlated with rs2305619 SNP (R^2^ = 0.7 and R^2^ = 0.9).

**Table 2:** Details of *PTX3* SNPs.

SNP ID(rs)	Position(bp)	Location	HWEChi-square test*(p-value)*	Minor allele	Minor allelefrequency	Minor allele frequency(HapMap)	R^2^
**rs2305619**	157154861	Intron1	*0.97*	A	0.48	0.508	reference
**rs3816527**	157155314	Exon 2	*0.32*	C	0.43	0.403	0.7
**rs1840680**	157156029	Intron2	*0.98*	A	0.48	0.491	0.9

HWE, Hardy-Weinberg Equilibrium test.

R^2^, Correlation coefficient.

We compared genotype and allele frequencies of the three SNPs and the haplotype frequencies in AMI patients and controls or angiographically controlled individuals. None of the genotypes (Table 3), alleles or haplotypes (not shown) were significantly associated with the risk of AMI.

**Table 3:** Distribution of rs2305619, rs3816527 and rs1840680 SNP genotypes in AMI patients and controls.

SNP	Genotype	AMI (1751)	Controls (1494)	*p-value*
**rs2305619**	**AA**	400 (22.94%)	359 (24.03%)	*0.68*
	**AG**	871 (49.94%)	746 (49.93%)	
	**GG**	473 (27.12%)	389 (26.04%)	
**rs3816527**	**CC**	310 (17.74%)	292 (19.58%)	*0.39*
	**AC**	864 (49.46%)	714 (47.89%)	
	**AA**	573 (32.80%)	485 (32.53%)	
**rs1840680**	**AA**	395 (22.75%)	355 (23.76%)	*0.72*
	**AG**	867 (49.94%)	746 (49.93%)	
	**GG**	474 (27.30%)	393 (26.31%)	

### Association between *PTX3* Genetic Variants and PTX3 Plasma Levels

**Figure 2:**
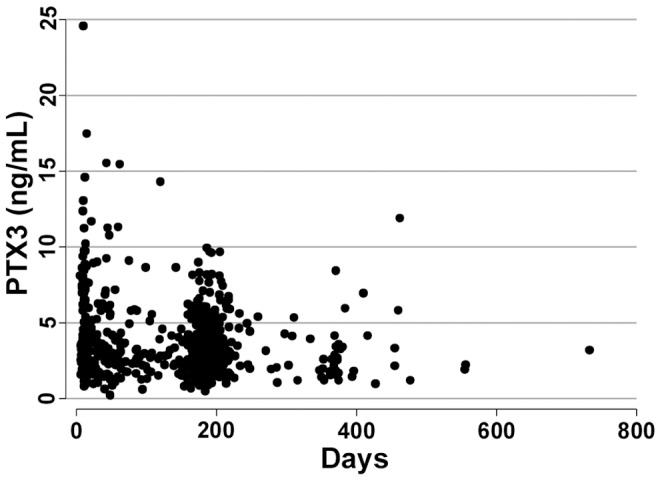
Distribution of PTX3 levels in 704 AMI patients in relation to the time of blood collection. Blood samples were collected from 5 to 733 days after the AMI. This range was divided into tertiles: the first from 5 to 63 days, the second from 64 to 184 days and the third from 185 to 733 days. The mean PTX3 concentration was calculated for each tertile.

We examined the effects of the three SNPs on PTX3 plasma levels in the two groups, measuring PTX3 in 1368 subjects, 704 AMI patients and 664 controls, not selected for any baseline characteristics or genotype (data not shown). PTX3 plasma levels (mean ± SD) adjusted for age and sex were significantly higher in AMI patients (3.61±2.43 ng/mL) than controls (2.93±1.52 ng/mL, p = 0.0001). Figure 2 reports the distribution of PTX3 levels according to the time of blood collection for AMI patients, divided into three intervals (tertiles). For each tertile, the mean PTX3 concentration was calculated. In the first tertile, the level (4.18±3.24 ng/mL) was significantly different (p<0.0001) from the second (3.31±1.79 ng/mL) and the third (3.33±1.89 ng/mL). No differences were found in the distribution of the rs2305619, rs3816527 and rs1840680 SNPs in relation to the time of blood collection (p = 0.38, p = 0.27 and p = 0.42, respectively).

Table 4 reports the distribution of PTX3 plasma levels among rs2305619, rs3816527 and rs1840680 genotypes. The differences were significant only in controls: AA carriers for rs2305619 (vs. AG and GG genotypes) and AA carriers for rs1840680 (vs. AG and GG genotypes) had higher PTX3 levels. For rs3816527, plasma PTX3 concentrations were higher in AC carrier cases and CC carrier controls.

**Table 4:** Plasma PTX3 distribution among SNP genotypes in AMI patients and controls.

		AMI (704)		Controls (664)	
			PTX3 (ng/mL)		PTX3 (ng/mL)
**SNP**	**Genotype**	**No.**	**Mean (SD)**	**No.**	**Mean (SD)**
**rs2305619**	**AA**	154	3.84 (2.58)	152	3.22 (1.60)
	**AG**	344	3.67 (2.54)	352	2.84 (1.49)
	**GG**	206	3.32 (2.11)	160	2.83 (1.47)
	***p-value***		*0.09*		*0.02*
**rs3816527**	**CC**	118	3.56 (2.07)	126	3.22 (1.54)
	**AC**	344	3.80 (2.74)	331	2.90 (1.54)
	**AA**	242	3.36 (2.10)	207	2.79 (1.46)
	***p-value***		*0.16*		*0.02*
**rs1840680**	**AA**	154	3.89 (2.60)	149	3.25 (1.61)
	**AG**	340	3.66 (2.54)	355	2.83 (1.49)
	**GG**	210	3.31 (2.10)	160	2.83 (1.47)
	***p-value***		*0.06*		*0.009*

p-value from F test (ANOVA).

**Figure 3:**
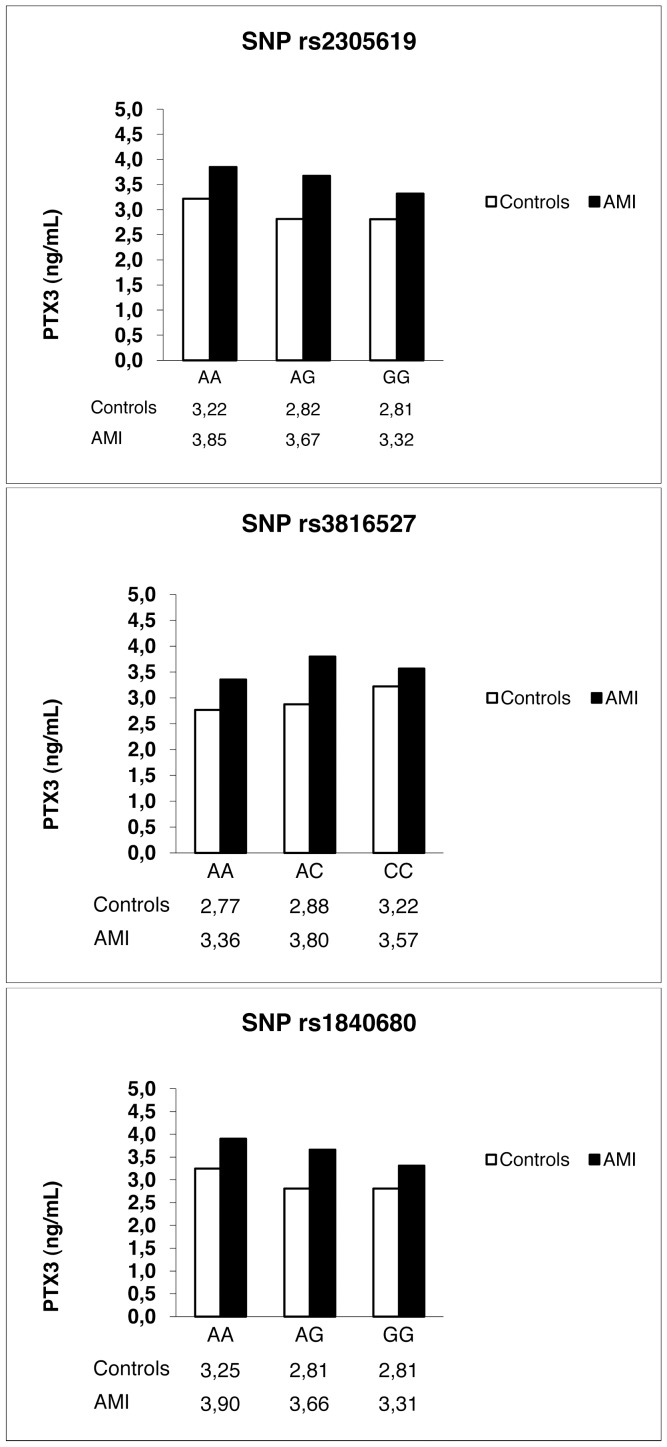
Histograms of the mean PTX3 levels in AMI patients and controls in relation to genotype. Each graph shows the mean PTX3 levels (ng/mL), adjusted for sex and age, in AMI patients and controls according to rs2305619, rs3816527 and rs1840680 genotypes.

Fitting a linear regression model adjusted for age and sex, the genotype and the diagnosis (AMI or controls) were significantly associated with PTX3 levels for all three SNPs (p = 0.0083 for genotype and p<0.001 for diagnosis, p = 0.0347 and p<0.001, p = 0.0032 and p<0.001, respectively, for rs2305619, rs3816527 and rs1840680). The interaction between diagnosis and genotype was not significant for the three SNPs, suggesting an association of *PTX3* SNPs with PTX3 levels independent of the pathological condition (Figure 3).

We examined the possible association between PTX3 plasma levels and haplotype frequencies in AMI patients and controls. Like each individual SNP, haplotypes were associated with different PTX3 plasma levels (overall p-value = 0.01, Table 5). Additive values estimated the change in the expected PTX3 plasma levels due to a given haplotype compared to the most common GAG haplotype, taken as reference. ACA and AAA haplotypes (for rs2305619, rs3816527 and rs1840680) were associated with significantly higher PTX3 levels. The ACA haplotype (frequency 42%) was associated with an increase of 0.05 ng/mL in PTX3 plasma levels, and the increase was 0.08 ng/mL for the AAA haplotype (frequency 5%) compared with the GAG haplotype (frequency 52%). These two effects were observed in AMI patients and controls (heterogeneity test: p = 0.46 for the AAA haplotype and p = 0.30 for GAG).

**Table 5:** Common haplotypes of the SNPs rs2305619, rs3816527 and rs1840680 and PTX3 plasma levels.

Haplotype	Count	Frequency	Additive valuePTX3 (ng/mL)	95% CIPTX3 (ng/mL)
**GAG**	1427	0.52	reference	reference
**ACA**	1162	0.42	0.05	0.009–0.085
**AAA**	138	0.05	0.08	0.009–0.158

### Association between PTX3 Plasma Levels and Prognosis

We assessed the influence of *PTX3* genetic variants on the prognosis of AMI patients. Among the 1448 AMI patients from the GISSI-Prevenzione trial, 120 deaths (8.29%), 32 cardiovascular deaths (2.21%) and 64 reinfarctions (4.42%) occurred during the three-year follow-up. Blood for PTX3 assay was available for 68 of the 120 deaths, 19 of the cardiovascular deaths and 32 of the reinfarctions. Patients with an event occurring within three years after the index event were significantly older (p<0.0001 for death, p = 0.002 for cardiovascular death, p = 0.02 for reinfarction) than the event-free ones. Cardiovascular deaths were significantly more frequent among females (p = 0.03). Other clinical covariates, and SNP genotypes, were not associated with events. After adjustment for age and sex, PTX3 levels were significantly associated with the risk of all-cause death (p = 0.02, OR 1.10, 95% CI: 1.01–1.20), but not with cardiovascular death (p = 0.09, OR 1.12, 95% CI: 0.98–1.27) or reinfarction (p = 0.67, OR 1.03, 95% CI: 0.90–1.18). The distribution of the events did not differ (p = 0.59 for death, p = 0.94 for cardiovascular death and p = 0.59 for reinfarction) among the three groups identified according to the time of blood collection (Figure 2).

## Discussion

The *PTX3* gene encodes for the long pentraxin PTX3, a molecule involved in the inflammatory response, with possible cardio- and athero-protective functions [20], [21]. Multiple genetic and environmental factors are believed to increase susceptibility to AMI. We found that three common *PTX3* polymorphisms (rs2305619, rs3816527 and rs1840680) did not significantly influence the risk of AMI in an European population, even if these SNPs, and the corresponding haplotypes, are associated with different levels of PTX3 in the blood. We also found a correlation between PTX3 plasma levels and all-cause death subsequent to AMI.

The absence of association between *PTX3* genetic variants and the risk of AMI is very likely to be a true result since the sample was adequately powered to test for the planned comparisons.

The mechanism by which *PTX3* SNPs affect PTX3 plasma levels has still to be clarified but possibly rs2305619, rs3816527 and rs1840680 belong to a larger haplotype block than the one considered here or else they are in linkage with a genetic variant located in a regulatory region, perhaps the *PTX3* promoter. In such a position, a polymorphism could affect the binding of a transcription factor to its consensus binding site, modifying the PTX3 expression level. It cannot be completely ruled out that *PTX3* SNPs are related to high plasma levels of a functionally less active protein. The second SNP we studied (rs3816527) causes an amino acid change (Asp48Ala) in a strategic position of the PTX3 primary structure. This could potentially interfere with the N-terminal-mediated binding of PTX3 to its ligands. Moreover, amino acid 48 is located between the two cysteine residues in positions 47 and 49 involved in the formation of the inter-chain disulfides required for the tetrameric arrangement of four PTX3 protomers [29].

Different levels of PTX3 in the blood, depending on genetic variants, may influence the outcome of certain infections. The GAG haplotype is associated with a protective effect against *Mycobacterium tuberculosis* [22] or *Pseudomonas aeruginosa* colonisation [23]. However, these two studies did not look into the correlation between PTX3 levels and *PTX3* haplotypes. We found that the most frequent haplotype, GAG, associated with lower plasma PTX3 levels than ACA and AAA. Conceivably haplotypes affecting PTX3 levels are not relevant for the risk of AMI. The minor allele of rs2305619 has been found significantly associated with higher plasma PTX3 levels and with disease severity in lung-transplant patients with idiopathic pulmonary fibrosis [30].

We found a mean of 2.93±1.52 ng/mL circulating PTX3 in controls. This is significantly different from the mean in AMI patients (3.61±2.43 ng/mL, p = 0.0001), even when blood was sampled at the earliest 5 days after the AMI event, the interval previously reported to be needed for PTX3 levels to return to normal [18]. For the AMI patients, we analyzed the distribution of PTX3 plasma levels in three intervals after the event. Up to three months, the mean PTX3 plasma concentration was higher (4.18±3.24 ng/mL) than in a later period (3.32±1.89 ng/mL, from three months to two years), when the levels are similar to those in healthy subjects. This suggests that the high PTX3 levels from 5 to 64 days after the event probably explain the significant difference in levels between our AMI patients and controls. These results suggest that in AMI patients, in a few days after the onset of symptoms, PTX3 plasma levels return to values that, while still in the normal range, remain higher than in healthy subjects for at least three months.

Our data also confirm the prognostic value of PTX3 plasma levels in AMI patients. PTX3 was an independent predictor of three-month mortality, compared with other biomarkers considered reliable predictors of death in these patients [19], [31], [32]. We found a correlation between PTX3 plasma levels and all-cause death after AMI. We did not see any correlation between *PTX3* genetic variants and susceptibility to AMI, despite the strong association between *PTX3* SNPs and PTX3 plasma levels. Neutral results regarding the association between *PTX3* genetic variants and AMI are very likely to be true. However, given the rather large effect size (OR at least 1.19) detectable by our sample size, we cannot exclude the presence of a more modest association. Similar data were obtained for another member of the PTX superfamily: C-reactive protein, like PTX3, binds LDL [33] and is present in the atherosclerotic plaque [34]. Several epidemiological studies have reported a relation between high basal CRP levels and CAD risk [35], [36]. These observations suggest a causal role of CRP in CAD. Several SNPs have been identified in the *CRP* gene. Independently or combined in haplotypes, some of these were associated with different CRP plasma levels. A meta-analysis of genetic studies involving a new Bayesian method (permitting integration of information across studies that had typed a partially overlapping set of *CRP* SNPs) has provided evidence of four functional SNPs at the *CRP* locus that influence its circulating concentration [37]. The relationship between genetically determined CRP blood levels and the risk of CAD has been debated for years. Genetic studies based on the “Mendelian randomisation” approach have assessed the causal relevance of CRP itself to coronary heart disease. Large case studies suggest that the CRP concentration itself is unlikely to be even a modest causal factor in CAD [38], [39].

Given our results on PTX3 and the evidence on CRP in relation to CAD risk, we envision a similar situation for PTX3 and AMI risk. This study highlights the effect of *PTX3* SNPs on PTX3 plasma levels which peak in patients with AMI but then remain higher than in controls for at least three months. We found no significant association between *PTX3* SNPs and the risk of AMI. PTX3 is a prognostic marker after AMI but genetically determined high PTX3 levels do not seem to influence the risk.
